# 2-Deoxyglucose and hydroxychloroquine HPLC-MS-MS analytical methods and pharmacokinetic interactions after oral co-administration in male rats

**DOI:** 10.21203/rs.3.rs-2675386/v1

**Published:** 2023-03-14

**Authors:** Dongxiao Sun, Sangyub Kim, Deepkamal Karelia, Yibin Deng, Cheng Jiang, Junxuan Lü

**Affiliations:** Penn State College of Medicine; Penn State College of Medicine; Penn State College of Medicine; University of Minnesota; Penn State College of Medicine; Penn State College of Medicine

**Keywords:** HPLC-MS-MS, oral drug-drug PK interference, drug-drug interaction, 2-deoxyglucose, hydroxychloroquine

## Abstract

Our previous work has shown a synergistic tumoricidal action of the hexokinase (HK) inhibitor 2-deoxyglucose (2-DG) and the autophagy inhibitor chloroquine (CQ) on HK2-addicted prostate cancers in animal models through intraperitoneal injections. Here we developed high performance liquid chromatography-tandem mass spectrometry (HPLC-MS-MS) methods for 2-DG and clinically favored drug hydroxychloroquine (HCQ) and explored PK interaction of the orally administered drugs in a jugular vein cannulated male rat model, which allowed serial blood collection before and 0.5, 1, 2, 4 and 8 h after a single gavage dose of each drug alone or simultaneously after appropriate washout periods between the drugs. The results demonstrated a rapid and satisfactory separation of 2-DG standard from common monosaccharides by HPLC-MS-MS multi-reaction monitoring (MRM) and the presence of endogenous “2-DG”. Application of the HPLC-MS-MS 2-DG and HCQ methods to sera samples of 9 evaluable rats showed a peak time (*T*_*max*_) of 2-DG of 0.5 h after 2-DG dosing alone or with HCQ and glucose-like PK behavior. With a seemingly bi-modal time course for HCQ, the *T*_*max*_ for HCQ dosing alone (1.2 h) was faster than that for the combination (2 h; p = 0.013, 2-tailed t-test). After combination dosing, the peak concentration (*C*_*max*_) and area under the curve (*AUC*) of 2-DG were decreased by 54% (p < 0.0001) and 52%, whereas those for HCQ were decreased by 40% (p = 0.026) and 35%, respectively, compared to single dosing. The data suggest significant negative PK interactions between the two oral drugs taken simultaneously and warrant optimization efforts for the combination regimen.

## Introduction

2-Deoxyglucose (2-DG) is a synthetic glucose analog in which the 2-hydroxyl group is replaced by a hydrogen atom. 2-DG, like glucose, enters the glucose-loving cancer cells through the glucose transporter and is phosphorylated by a hexokinase (HK), the first and rate-limiting enzyme in glycolysis. Not further metabolizable, 2-DG-6-phosphate accumulates in cells and competitively inhibits HK and glycolysis, leading to cancer cell cycle arrest and autophagy, which promotes cell survival through antagonizing apoptosis (1). Due to its cytostatic nature, 2-DG monotherapy has little efficacy in early stage human clinical trials for the treatment of cancer including prostate cancer (2).

For decades, chloroquine (CQ) and hydroxychloroquine (HCQ) are orally available drugs for preventing and treating malaria caused by mosquito bites (3). Known as lysosomotropic autophagy inhibitors, they are also indicated for treating and managing autoimmune diseases such as lupus and rheumatoid arthritis (4). Based on the rationale that an induction of autophagy in HK2-addicted prostate cancer cells by 2-DG counteracts against their cell death by apoptosis, our previous work has shown a synergistic tumoricidal action of the 2-DG and CQ combination through intraperitoneal injections in several prostate cancer animal models (1). Because 2-DG exposure of prostate cancer cells took between 4 to 6 h to activate AMP-activated protein kinase (AMPK) that drove the autophagy induction, the animal daily dosing regimen (Monday – Friday) was based on such a signaling consideration and carried out with 2-DG injection in the morning and CQ injection in the afternoon (1).

Pharmacokinetic (PK) interaction is one of the main drug-drug interactions (DDIs) and a major cause of medication error (5). Given that cancer patients receive multiple concomitant treatments, such as chemotherapy, radiotherapy, hormonal therapy, and supportive care agents, they are at high risk for DDIs. The oral administration of the cancer treatment has become common over decades because of availability of oral anticancer agents and substantial benefits of the use, including convenience, no needle injury, self-medication, low infection risk, and high patient compliance. Since 2-DG and HCQ are taken by human patients orally, knowledge of their DDIs will inform the optimal dosing regimen to achieve therapeutic benefit over harm. Whereas the PK behavior of each drug has been extensively documented in rodents and humans, their PK interactions, if any, after simultaneous oral administration have not been reported.

## Theoretical

Methodologically, different instrumental protocols have been reported for 2-DG previously. One of the early methods for determining the purity of 2-DG preparations was gas chromatography. Because 2-DG is a nonvolatile, high melting point solid, it was made more volatile by derivatization using N-trimethylsilylimidazole in pyridine (6). Another method analyzed the presence of tritiated ^3^H-2-DG in rat muscle using chromatography-radioisotopic (RI) detection (7). Yet another non-derivatization measurement of 2-DG in topical formulations used high performance liquid chromatography (HPLC)-ultraviolet detection (UV) at 195 nm (8), due to the lack of a chromophore absorbing above 200 nm in 2-DG. The analytical columns included a μBondapak 10 μm NH_2_ column and a Varian Micropak 10 μm NH_2_ column. As 2-DG has a very short retention time on these columns, plus the low sensitivity and selectivity of UV detection, the HPLC-UV method performed poorly at separating 2-DG from the highly abundant glucose and other structurally related monosaccharides in blood samples. The development of fluorescence detection a decade ago enhanced the sensitivity for 2-DG analysis through derivatization with 2-aminobenzoic acid in the presence of sodium cyanoborohydride at 80°C for 45 min (9). The subsequent separation by HPLC and detection by fluorescence took an additional 1 h for each sample. The serious drawbacks of the fluorescence method included the extra reaction steps, the long HPLC time per sample and not directly measuring the actual analyte (9). In summary, no direct method with high sensitivity, selectivity and operational efficiency has been reported for 2-DG quantitation in biological fluid samples.

In contrast, HPLC with tandem mass spectrometry (HPLC-MS-MS) has been used for detection of CQ-family drugs, including hydroxychloroquine (HCQ) and its major metabolites (10). HPLC-MS-MS is a powerful analytical technique that combines the separating power of HPLC with the highly sensitive and selective mass analysis capability of triple quadrupole MS.

Herein, we developed a new HPLC-MS-MS method for the separation and quantification of 2-DG in rat serum and refined the HPLC-MS-MS detection of HCQ. We applied these analytical protocols for 2-DG and HCQ drug-drug PK interactions in a jugular vein cannulated rat model, which afforded serial PK blood collections following the gavage administration of each drug alone or their combination.

## Methods

### Materials

Hydroxychloroquine sulfate (HCQ) was purchased from TCI Chemicals (Portland, OR, USA) (catalog H1306). Deuterated hydroxychloroquine-d4 (HCQ-d4) were purchased from Toronto Research Chemicals (Toronto, ON, Canada) as the analytical internal standard (IS). 2-Deoxy-D-glucose (2-DG) was purchased from Sigma-Aldrich (St. Louis, MO, USA) (catalog number D6134). ^13^C_6_-2-deoxy-D-glucose (^13^C_6_-2-DG) and ^13^C_1_-2-DG (in initial method testing) were purchased from Toronto Research Chemicals (Toronto, ON, Canada) as IS for 2-DG. Formic acid was purchased from J. T. Baker (Phillipsburg, New Jersey, USA). Optima LC-MS grade water, acetonitrile and methanol and other chemicals were purchased from Fisher Scientific (Fair Lawn, New Jersey, USA).

### HPLC-MS-MS analysis method for 2-DG

A new method was developed using Sciex 6500 + QTrap MS coupled with an ExionLC separation system (Waltham, MA, USA) and was able to separate 2-DG from other simple sugars including D-glucose, fructose, mannose, and galactose (Fig. S1). A Luna 3 μm NH2 analytical column (2 × 100 mm, Phenomenex, Torrance, CA, USA) was used. The isocratic elution was carried out using a flow rate of 0.5 mL/min with water as mobile phase A (17%) and acetonitrile as mobile phase B (83%). The column was kept at 30 °C during the separation procedure.

The Sciex 6500 + Q Trap mass spectrometer was equipped with an electrospray ionization probe operated in negative mode. The decluster potential (DP) was – 30 V; the entrance potential (EP) was – 10 V, the collision energy (CE) was – 12 V for 2-DG and – 19 V for ^13^C_6_-2-DG, and the collision cell exit potential (CXP) was – 11 V for 2-DG and – 10 V for ^13^C_6_-2-DG. The curtain gas (CUR) was 35 L/h, the collision gas (CAD) was medium. The ion spray voltage was – 4500 V, the temperature was 300 °C. The flow rate for gas 1 was 25 L/h and for gas 2 was 25 L/h. The multiple reaction monitoring mode (MRM) was used to analyze and quantify 2-DG, with the transitions of *m/z* 163 > 85 for 2-DG and 169 > 89 for ^13^C_6_-2-DG. All peaks were integrated and quantified by Sciex OS 1.5 software. The quantification limit (signal/noise > = 10) for 2-DG was 10 ng/mL, and the detection limit (signal/noise = 3) was 2.5 ng/mL.

### HPLC-MS-MS analysis method for HCQ

An EXionLC separation system with a 1.7 μm Acquity UPLC BEH C18 analytical column (2.1 × 50 mm, Waters, Dublin, Ireland) was used to separate HCQ from other serum constituents. Gradient elution was conducted using a flow rate of 0.3 mL/min with the following conditions: initiate at 2% mobile phase B (acetonitrile) and 98% mobile phase A (0.1% formic acid in water), and linear gradient to 98% mobile phase B in 2 minutes, and keep the mobile phase B at 98% for another 2 minutes to flush the column before back to the initial conditions to equilibrate the column.

HCQ was analyzed using a Sciex 6500 + Q Trap mass spectrometry, equipped with an electrospray ionization probe operated in positive mode. The decluster potential (DP) was 70 V for; the entrance potential (EP) was 10 V, the collision energy (CE) was 32 V and the collision cell exit potential (CXP) was 15 V, while the curtain gas (CUR) was 35 L/h, the collision gas (CAD) was set on high. The ion spray voltage was 4000 V, the temperature was 300 °C. The flow rate for gas 1 and gas 2 was 25 L/h, respectively. The MRM was used to analyze and quantify HCQ and its internal standard HCQ-d4, with the transitions of *m/z* 336 > 247 for HCQ and *m/z* 340 > 251 for HCQ-d4. All peaks were integrated and quantified by Sciex OS 1.5 software. The quantification limit (signal/noise > = 10) for HCQ was 0.1 ng/mL, and the detection limit (signal/noise = 3) was 0.025 ng/mL.

### PK experiments: serial blood collection after 2-DG, HCQ single oral dose or combo dose

The animal work had been conducted with the approval of the Institutional Animal Care and Use Committee of Penn State College of Medicine, Hershey, PA campus. Jugular vein cannulated CD male rats (200–300 g) were purchased from Charles River, Wilmington, MA. They were housed individually to prevent damage to the catheter. They were provided free access to water and rodent chow pellets. After quarantine and acclimation, they were used in the PK dosing sequence as shown in Table 1. The 2-DG was gavage-administered at 372 mg/kg body weight. HCQ was gavaged at 124 mg/kg body weight. These dosages were based on our early mouse efficacy studies (1) with inter-species allometric dose conversion adjustment. The combo dosing was delivered sequentially within 2 minutes of each other.

On each day of experiment, dosing started the PK clock as 0 h. Sequential blood collection (approximately 0.3 ml per time point) were performed at the indicated time points ± 5 minutes (Table 1), followed by proper washout periods based on prior knowledge of PK behaviors of each drug. Serum (no heparin or EDTA anticoagulant) samples were stored at −80°C for later analyses by LC-MS-MS-multiple reaction monitoring (MRM) as detailed above. Nine sets of complete data were evaluable for the results (n = 9 rats). The data were plotted graphically as timepoint mean ± SEM vs. blood collection time post dose.

### Serum sample preparation procedure for 2-DG measurement

A stock solution of 2-DG was prepared in water and serially-diluted into working solutions of 25 – 10,000 ng/mL. ^13^C_6_-2-DG was dissolved in DMSO at 2.5 mg/mL as stock solution and was diluted to 25,000 ng/mL by methanol as the working solution. All solutions were kept at –20°C before use. Standards were made by spiking 5 μL 2-DG standard working solution and 5 μL ^13^C_6_-2-DG internal standard working solution into 40 μL acetonitrile / methanol (50/50) with final concentrations of 25 ng/mL to 10,000 ng/mL. Serum samples were processed through organic solvent extraction. After spiking 5 μL ^13^C_6_-2-DG working solution into 5 μL serum, followed by adding 40 μL acetonitrile/methanol (50/50) to precipitate proteins by votexing and subsequent centrifugation at 8765× *g* for 10 min at 4°C. The supernatant was transferred to HPLC vials before loading onto HPLC-MS-MS system.

### Serum sample preparation procedure for HCQ measurement

A stock solution of HCQ was prepared in water and diluted into working solutions of 10 – 5,000 ng/mL. HCQ-d4 working solution was prepared in water at 100 ng/mL. All solutions were kept at –20°C before use. For standard curve, 5 μL HCQ serially-diluted working solutions and 5 μL HCQ-d4 working solution were spiked into 10 μL blank serum. Then, 30 μL methanol containing0.1 % formic acid was added to precipitate the proteins. After vortexing and subsequent centrifugation at 8,765 *g* for 10 minutes at 4°C, the supernatant was loaded to HPLC-MS-MS system, with final concentrations of 1 ng/mL to 500 ng/mL for the HCQ linear standard curve. Standard curves were constructed by plotting the ratio of peak area of analyte to peak area of the corresponding IS versus the nominal analyte concentration.

Serum samples were processed the same way as that for standard samples: 5 μL HCQ-d4 working solution was spiked into 10 μL serum, and 35 μL methanol containing .1 % formic acid was added to precipitate the proteins before the supernatant was loaded onto the HPLC-MS-MS system.

### Data presentation and Statistical analyses

For graphical visualization, timepoint group mean and SEM were plotted against blood collection time. Peak time *T*_*max*_ and peak drug concentration *C*_*max*_ were determined based on individual rat data and compared between single dosing vs. combo dosing by 2 tailed t-test and a variance setting that was appropriate to the dataset. Area under the curve *AUC*_*0 – xh*_ was estimated based on timepoint mean data plots (by weighing paper cutout to 0.1 mg). Post-peak half-life *t*_*1/2*_ was estimated based on semi-log plots of timepoint group mean data.

## Results

### Novel HPLC-MS/MS analysis method for 2-DG

**Figure 1:**
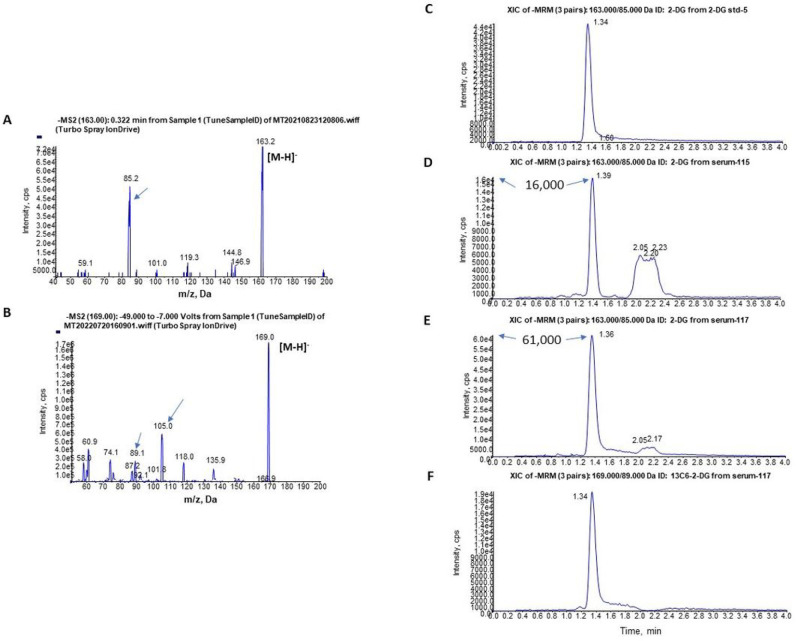
Mass spectra of (A) 2-DG standard and (B) ^13^C_6_-2-DG internal standard (IS) and chromatograms for standard/IS and serum samples (C-F). A. Product ion scan for 2-DG standard; B. product ion scan for ^13^C_6_-2-DG as internal standard (IS); HPLC-elution MRM profiles of C. 2-DG standard; D. 2-DG in pre-dose (control) serum; E. 2-DG in post-dose (1 h treated) serum; F. ^13^C_6_-2-DG (IS) in post-dose (1 h treated) serum.

For the analysis of 2-DG by HPLC-MS/MS, negative mode was applied due to the molecular structure and chemical property of 2-DG and its internal standard (IS) ^13^C_6_-2-DG. By infusion under Q1 scan analysis, [M-H]^–^ was found at *m/z* 163 for 2-DG (Fig. 1A) and *m/z* 169 for IS ^13^C_6_-2-DG. By product ion scan, a major fragment of 2-DG was found at *m/z* 85 (Fig. 1A) and *m/z* 163 → 85 transition was used for MRM. For IS ^13^C_6_-2-DG fragments at *m/z* 105 and *m/z* 89 were found (Fig. 1B). As *m/z* 169 > 89 showed higher density MRM peak than *m/z* 169 > 105, *m/z* 169 → 89 transition was selected for MRM of the IS.

Because of structural similarities with many simple sugars such as glucose, fructose, mannose, galactose, etc. the separation of 2-DG from these sugars, especially the highly abundant glucose, in serum is always difficult. A Luna 3 μ NH_2_ column (2.0 × 100 mm) was chosen for the separation here. The normal phase column was flushed by isopropanol for hours to change it to a reversed phase column for more mobile phase selections. After careful optimization, an isocratic program of water:acetonitrile (17:83) was used to successfully separate 2-DG from other monosaccharides (Fig. S1). MRM profiles of 2-DG standard (Fig. 1C) and ^13^C_6_-2-DG IS (Fig. 1F) showed single peak over baseline.

However, an endogenous peak was detected to elute at the same retention time as that of 2-DG standard, as well as with identical molecular ion and fragment (*m/z* 163/85) with 2-DG in pre-dose (control) serum (serum-115) (Fig. 1D), and it was separated from other sugars (retention time 2–2.4 min). The chromatograph pattern of 1 h post dose serum sample from the same 2-DG-treated rat (serum-117) (Fig. 1E) showed sharply increased peak intensity at the same retention time with 2-DG standard and the endogenous peak only (also see Fig. S2). This pattern was recapitulated by spiking 2-DG into the pre-dose control serum (Fig. S3). Because of presence of the endogenous “2-DG” peak in the pre-dose serum, the 2-DG working standard solutions were therefore made in water instead of in a blank control serum, with linear range of 25 ng/mL to 10,000 ng/mL for the 2-DG standard curve.

Since the rats ate laboratory rodent chow pellets made of practical feed ingredients, the endogenous “2-DG” peak in the pre-dosing control serum could be an isomer(s) of 2-DG, possibly a deoxy sugar. Biologically important deoxy sugars include many examples, such as 6-deoxy-L-galactose, a constituent of cell membrane glycoproteins and glycolipids; 6-deoxy-L-mannose, which presents in plant glycosides; 6-deoxy-D-glucose, a natural product found in *Pogostemon cablin, Salmonella enterica,* and other organisms (https://lotus.nprod.net/). Separation of the exogenously dosed 2-DG from the endogenous deoxy sugar was not the focus of the current work. Future study may identify the component of the endogenous peak.

### HPLC-MS/MS analysis method for HCQ

**Figure 2:**
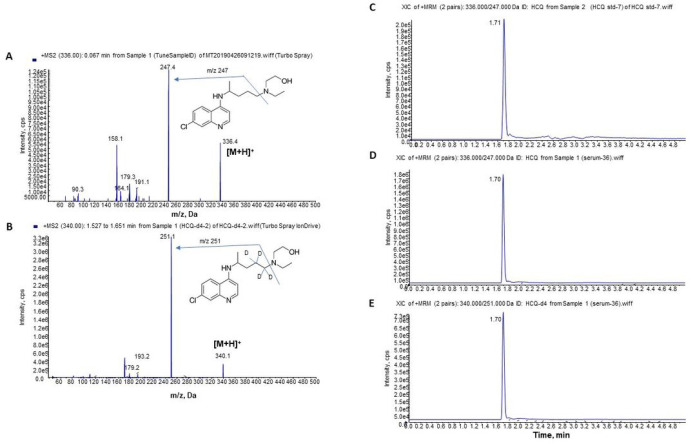
Mass spectra of (A) HCQ standard and (B) HCQ-d4 internal standard (IS) and chromatograms for standard/IS and serum samples (C-E). A. Product ion scan for HCQ standard; B. product ion scan for HCQ-d4 as internal standard (IS); HPLC-elution MRM profiles of C. HCQ standard; D. HCQ in post-dose (treated) serum; E. HCQ-d4 IS in post-dose (treated) serum.

To optimize MS conditions, ESI source in positive mode was applied and [M + H]^+^ was found at *m/z* 336 for HCQ (Fig. 2A) and *m/z* 340 for HCQ-d4 as IS (Fig. 2B) under Q1 scan analysis. MS/MS product ion scan for the fragmentation of the molecular ions detected specific product ion at m/z 247 for HCQ (Fig. 2A) and *m/z* 251 for HCQ-d4 (Fig. 2B). Thus, MRM transition of m/z 336 → 247 was selected for quantification of HCQ while *m/z* 340 → 251 was selected for quantification of IS HCQ-d4.

To achieve high resolution and efficiency for the analysis, the chromatography conditions were optimized for HCQ analysis with a five-minute gradient HPLC program using a 1.7 μm C18 column. Sharp peaks with clear baseline were achieved for HCQ standards (Fig. 2C) and HCQ-d4 IS(Fig. 2E). When different organic solvents were compared for the extraction of HCQ from serum, methanol plus 0. % formic acid was found to be the most efficient for a high recovery. Similar patterns were observed for HCQ and HCQ-d4 peaks in serum sample after extraction (Fig. 2D and 2E). The analytical method developed was specific for the analysis of HCQ in serum, showing no endogenous interfering components at the retention time of the analyte. The linear range of the method was 0.5–500 ng/mL for HCQ which covered the distribution of the concentrations from all serum samples.

### 2-DG PK metrics

**Figure 3:**
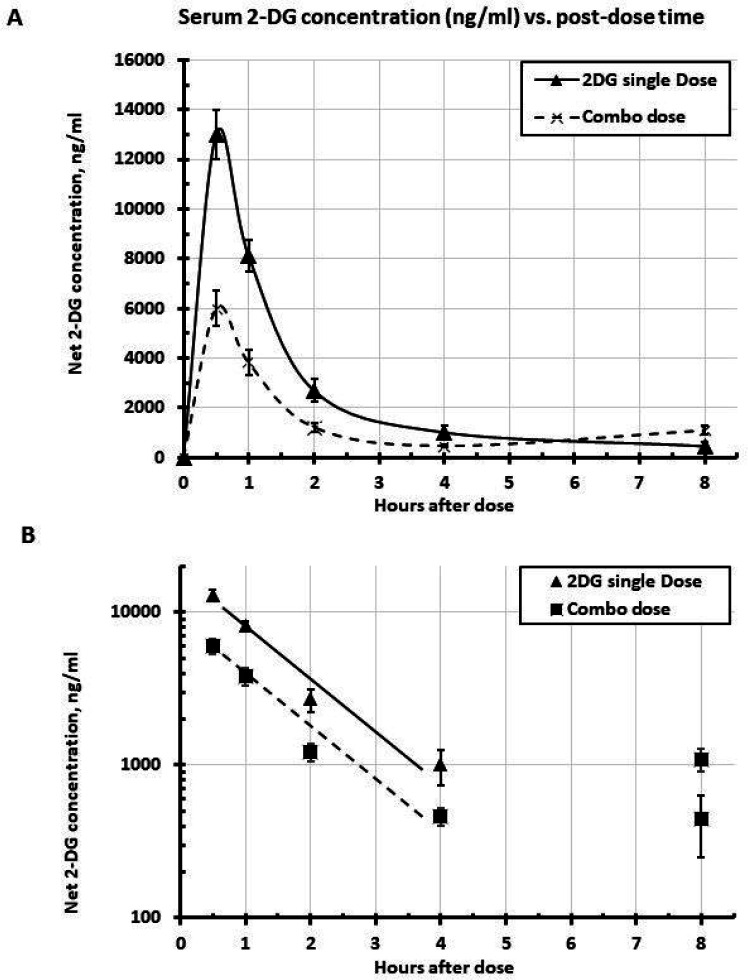
2-DG PK metrics: A. 2-DG serum concentration vs. time post single dose alone or combo with HCQ. B. Semi log plot, showing post-peak elimination slopes. Mean ± SEM, n= 9.

The newly developed method for measuring 2-DG in serum was used to analyze 2-DG levels across different time points. The endogenous “2-DG” peak detected in each pre-dose sample for single dose (902 ± 134 ng/ml) was not significantly different from that for combination dose (758 ± 66 ng/ml) (2-tailed t-test, p = 0.359). The net 2-DG concentrations for each rat were therefore obtained by subtracting the corresponding pre-dose “2-DG” value. The population PK curve was plotted as the timepoint mean of the group. When dosed alone, 2-DG (Fig. 3A, triangles, solid line) was taken up rapidly and peaked at 0.5 h and returned to pre-dose level by 2 h, as expected from glucose PK. However, when 2-DG and HCQ were dosed together, the *C*_*max*_ for 2-DG was decreased by 54% (2-tailed t-test, p = 0.000028) and the AUC_0 – 4h_ was reduced by 52% (Fig. 3, crosses, dashed line) (Table 2). Nevertheless, there was no change of the *T*_*max*_ (0.5 h) or the apparent post-peak elimination rate or half-life, *t*_*1/2*_ (Fig. 3B, see parallel lines with identical slopes in the semi-log plot) (Table 2).

### HCQ PK metrics

**Figure 4:**
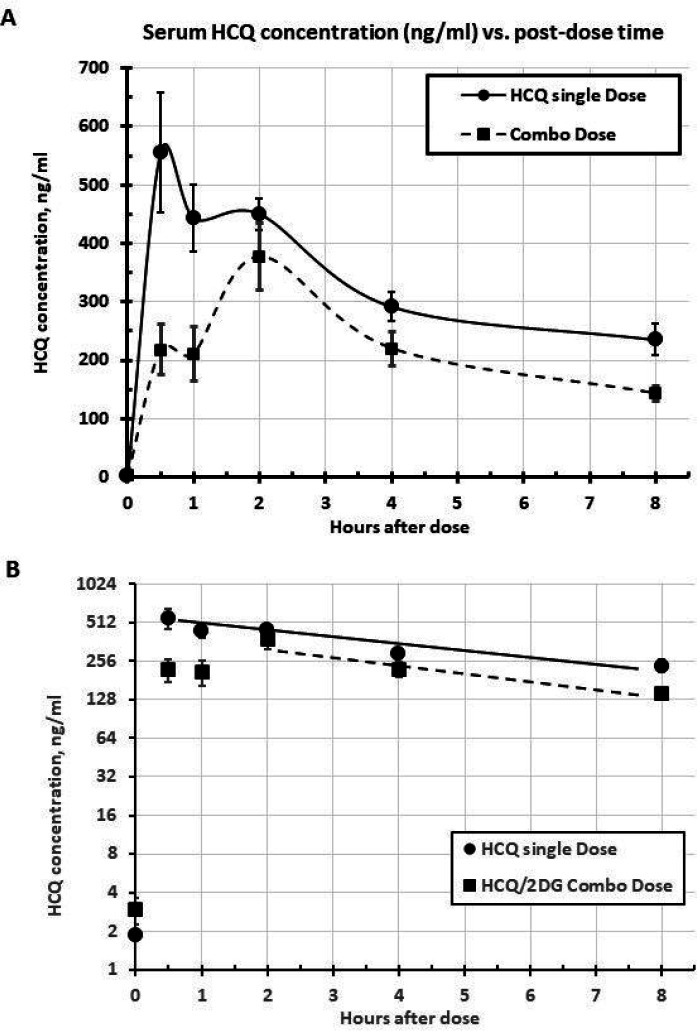
HCQ PK metrics: A. HCQ concentration vs time post single dose alone or combo with 2-DG. B. Semi log plot, showing post-peak elimination slopes. Mean ± SEM, n= 9.

The serum HCQ concentration vs. time profiles for the HCQ post single dose or combined with 2-DG are shown in Fig. 4A, with a seemingly bi-modal time course under each dosing condition. When HCQ was dosed alone (Fig. 4A, circles), *T*_*max*_ was on average 1.2 h (5 rats with 0.5 h; 4 rats with 2 h, out of 9 rats); *C*_*max*_ was on average 626 ng/ml (Table 2). The combo dosing with 2-DG (Fig. 4A, squares) resulted in a slower absorption with *T*_*max*_ = 2 h (all 9 rats at 2 h; 2-tailed t-test, p = 0.013) and *C*_*max*_ was lowered by 40% (377 ng/ml; 2-tailed t-test, p = 0.026) (Table 2). The cumulative HCQ exposure based on AUC_0 – 8h_ from combo dosing was decreased by approximately 35% (Table 2). The post-peak elimination rate (Fig. 4B, semi-log plot) was not affected by combo dosing, but did show much shallower slopes (therefore longer half-life *t*_*1/2*_) than that for 2-DG (Fig. 2B) (Table 2), as expected from previous HCQ PK information from the literature (3, 10).

## Discussions

### Methodological advantages

The HPLC-MS-MS methods reported above could measure 2-DG and HCQ in serum with high sensitivity, efficiency and resolution. The small volume of bio fluid (5–10 μL serum) needed is an advantage of these sensitive methods. Only 5 minutes running time for each method makes them ideal for higher-throughput bioanalysis as well as routine PK studies of each of these drugs. It is further noteworthy that the ability of the HPLC-MS-MS method for 2-DG to efficiently separate other monosaccharides from 2-DG lends the method for adaptation to studying these sugars with greater specificity in medicine and in many other fields.

### Implications for 2-DG and HCQ combination therapy of HK2-addicated cancers

Application of these methods to the rat sera samples indicated a mutual interference of the uptake/absorption between the two drugs if orally taken simultaneously, with a more profound effect of HCQ on 2-DG than *vice versa*. In retrospection, the daily dosing regimen of 2-DG in the morning and CQ in the afternoon used in our prostate cancer mouse models (1) inadvertently avoided the brunt of a negative DDI with the observed synergistic anti-cancer outcome. The rat PK interaction data warrant additional animal modeling work for optimization of dosing sequence to minimize the negative DDI in future human translation trials for therapy of HK2-addicted CRPC or cancers of same metabolophenotype in other organ sites.

### Implications for other human clinical indications

Beside treating malaria and certain autoimmune diseases clinically (4), HCQ is approved as a third line add-on drug for glycemic control in India for type II diabetes patients with consistent efficacy and remarkable safety for long term use (11, 12). Although improvement of circulating insulin level and tissue insulin sensitivity have been most often cited as its putative anti-hyperglycemia mechanisms, our observed negative PK interactions in the rat model suggest yet another and more direct mode of interaction in that HCQ decreases glucose absorption (by inference from 2-DG PK) in the gastrointestinal tract. Consistent with this speculation, two weeks of HCQ and rapamycin dual autophagy inhibitor treatment decreased 2-FDG PET-uptake from an *iv* dose (by inference glucose) by the tumor in sarcoma patients in Taiwan (13). In fact, hypoglycemia is a stated side effect of HCQ use in non-diabetic patients (Hydroxychloroquine Tablets: Package Insert / Prescribing Information - Drugs.com). In contrast to the global failure of HCQ for COVID-19 treatment, 2-DG has been shown in Phase II and Phase III trials in India to improve the outcome of COVID patients as much as a median reduction of 2.5 days to achieve normalization of specific vital signs parameters when compared with standard of care (14). How 2-DG and HCQ affect the absorption of the other at the gastrointestinal level awaits further investigation.

## Conclusions

The cutting-edge analytical methodologies of HPLC-MS-MS MRM enabled the assessment of PK metrics of 2-DG and HCQ in a rat model. The data suggest significant negative PK interactions between the two oral drugs taken simultaneously. The data warrant additional animal modeling work for optimizing their dosing sequence to minimize the negative DDIs in future human translation trials for cancer therapy or other indications.

## Figures and Tables

**Table 1:** Design of PK experiments.

			Blood draw time post dose					
Expt #	N of rats treated	Drug Treatment	0* h	0.5 h	1h	2h	4 h	8h
1	10	2-DG (gavage, 372 mg/kg)	x	x	x	x	x	x
	1-day washout							
2	10	HCQ (gavage, 124 mg/kg)	x	x	x	x	x	x
	3-day washout							
3	9	Combo (gavage, 372 mg 2-DG, 124 mg HCQ/kg	x	x	x	x	x	x

* Pre-dose baseline blood (~ 0.3 ml) was collected from each rat on the day before dosing started.

**Table 2:** Summary of PK metrics for gavaged 2-DG, HCQ each dosed alone or simultaneously.

	*2-DG metrics*				*HCQ metrics*			

Drug (dosage)	*T_max_*	*C_max_*	*AUC_0–4h_*	*t_1/2_*	*T_max_*	*C_max_*	*AUC_0–8h_*	*t_1/2_*

mg/kg	h	ng/ml	mg*	h	h	ng/ml	mg*	h

2-DG (372)	0.5 ± 0	13001 ± 977	153	0.8				

HCQ (124)					1.2 ± 0.3	627 ± 84	571	6.0

2-DG+HCQ (372+124)	0.5 ± 0	6006 ± 712	74	0.7	2.0 ± 0	377 ± 58	371	4.5
**[relative%]** **		**[46%]** **	**[48%]** **			**[60%]** **	**[65%]** **	

N (rats)	9	9			9	9		

P value, 2-tailed t-test	1	0.000028			0.013	0.026		

Mean ± SEM, N = 9.

* Based on weighing paper cutout to 0.1 mg.

** Percentage relative to respective single dose.

## Data Availability

The data that support the findings of this study are available in the supplementary material of this article
